# Commentary: Urinary Neopterin, a New Marker of the Neuroinflammatory Status in Amyotrophic Lateral Sclerosis

**DOI:** 10.3389/fnins.2021.645694

**Published:** 2021-03-23

**Authors:** Gisele Espíndola, Débora da Luz Scheffer, Alexandra Latini

**Affiliations:** ^1^Laboratório de Bioenergética e Estresse Oxidativo, Departamento de Bioquímica, Centro de Ciências Biológicas, Universidade Federal de Santa Catarina, Florianópolis, Brazil; ^2^Ambulatório de Doenças Neuromusculares e Neurogenéticas Hospital Universitário Polydoro Ernani de São Thiago, Universidade Federal de Santa Catarina, Florianópolis, Brazil; ^3^Programa de Pós-Graduação em Ciências Médicas, Universidade Federal de Santa Catarina, Florianópolis, Brazil

**Keywords:** inflammation, biomarkers, neurodegenerative diseases, amyotrophic lateral sclerosis, urine

Amyotrophic lateral sclerosis (ALS) is a heterogeneous progressive neurodegenerative disorder characterized by weakness and muscle atrophy in different areas of the body. The diagnosis is based on (i) the presence of signs of impairment of the lower and upper motor neurons in the cerebral motor cortex, brainstem, and spinal cord, (ii) electrophysiological evidence for chronic neurogenic changes, and on (iii) the exclusion of other diagnostic possibilities (Brooks, 1994; de Carvalho et al., 2008). The physiopathology of the disease is still not completely defined; however, chronic neuroinflammation is a hallmark of ALS (Takeda et al., 2020). So far, there is no cure or effective treatment for ALS and the lack of reliable biomarkers in peripheral biological fluids compromises the monitoring of the progression of the disease.

Lunetta et al. in an elegant work evaluated whether urinary neopterin levels could be used as a surrogate marker to predict the neuroinflammatory status of ALS (Lunetta et al., 2020). The authors found a negative association between the severity of the disease and urinary neopterin concentrations, claiming that those levels would represent the degree of inflammation in the nervous system of ALS patients. Indeed, neopterin levels have been used as a sensitive marker of immune system activation for decades (for a review see Ghisoni et al., 2015b). Neopterin is a byproduct of tetrahydrobiopterin (BH4) metabolism, which is stimulated under inflammation to generate more BH4 and enhance nitric oxide synthesis, catecholaminergic neurotransmitters production, and the metabolism of ether lipids (for a review see Ghisoni et al., 2015b). As stated by the authors, it has been traditionally understood that neopterin is formed and secreted by immune cells upon stimulation by inflammatory mediators, namely interferon gamma (IFN-γ) and interleukin 1 beta, hydrogen peroxide, and others (for a review see Ghisoni et al., 2015b); compounds known to be increased in the biological fluids of patients affected by ALS (Vu and Bowser, 2017; Jin et al., 2020).

Believed for decades to be an inert metabolic byproduct, the functional role and the origin of neopterin in the human nervous system are still not fully understood. The evidence available in the literature suggested that neopterin crosses the blood-brain barrier (BBB), and therefore, neopterin cerebrospinal fluid (CSF) levels might reflect peripheral neopterin concentrations (Fuchs et al., 1989). However, this process would occur at a very low quotient (1/40) (Hagberg et al., 1993), suggesting that CSF neopterin might have a local origin and be independently synthesized in the nervous system. This is supported by the lack of correlation between CSF and blood neopterin concentrations in patients with neurological-neuroinflammatory chronic conditions with normal BBB function (Kuehne et al., 2013). Additionally, our group recently demonstrated that neopterin is secreted by primary human brain cells, neurons, astrocytes and microglia, after being challenged with lipopolysaccharide or IFN-γ, supporting that neopterin CSF levels represent the central production of the compound (de Paula Martins et al., 2018). We also showed in experimental studies that intracellular neopterin has cytoprotective and memory enhancing effects mainly by activating *NRF-2*, the master regulator of cellular anti-oxidative responses (Moi et al., 1994; Itoh et al., 1999). The demonstrated capacity of neopterin to enhance the activity of the antioxidant system and the mitochondrial function to favor the anti-inflammatory facet of the immune system, and to facilitate the triggering of long-term potentiation–a molecular mechanism involved in hippocampal memory formation–allowed us to propose that neopterin is an endogenous cytoprotective compound with the specific role of increasing cellular resistance against stress; e.g., during chronic inflammatory conditions (Ghisoni et al., 2015a,b, 2016). This new data support that intracellular neopterin in non-immune cells is associated with cytoprotective functions, while increased levels of neopterin in peripheral fluids would likely represent the degree of inflammation. Therefore, the peripheral levels of this pterin might not necessarily reflect the local nervous system inflammatory status–that would require increased BBB permeability - but might instead reflect the degree of systemic activation of the inflammatory response.

It is widely understood that biomarkers are necessary for the development, testing, and ongoing positioning of new drugs and also for monitoring the evolution of a disease (*fda.org*). In this context, the quantification of neopterin levels in the urine has the potential value of revealing the degree of systemic inflammation. Indeed, Lunetta et al. showed a positive correlation between urinary neopterin levels and C-reactive protein concentrations (although not stated in Lunetta et al., 2020) it is assumed the levels were assessed in the blood). However, according to the box-plot shown in Lunetta's Figure 1 (Lunetta et al., 2020), 75% of ALS urinary samples shared similar levels of neopterin with the healthy control group, which compromise the use of urinary neopterin as a surrogate marker of the degree of neuroinflammation in this condition. Additionally, levels of urinary neopterin are more prone to show higher variability than in plasma, since they are shaped by age, retention time at the moment of sample collection, and others factors (Kampmann and Hansen, 1981). Although the sample size is small, Figure 1 shows the greatest variability of neopterin levels in the urine in young healthy adults, an age-group not included in Lunetta's work (Lunetta et al., 2020).

**Figure 1:**
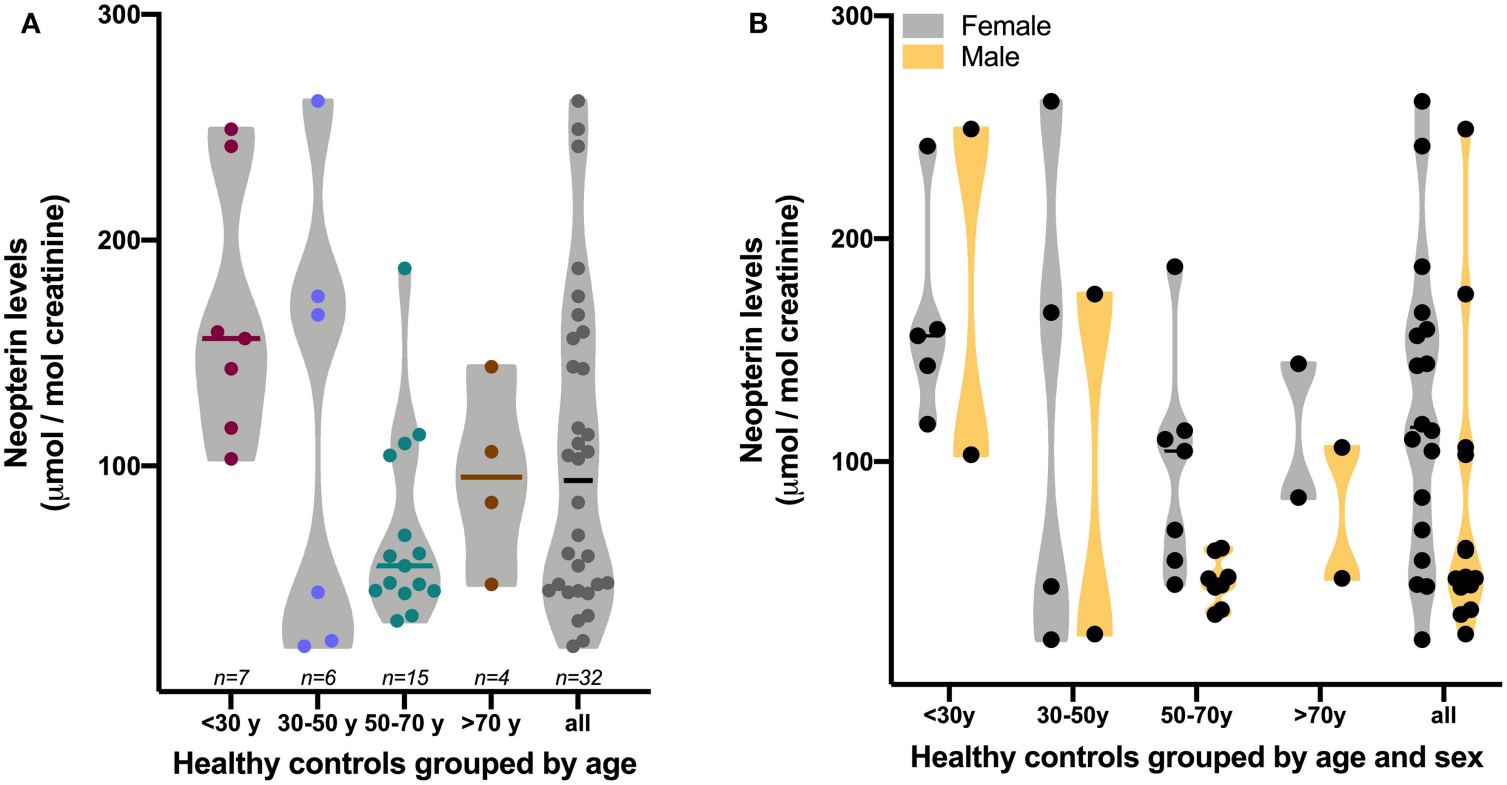
Levels of neopterin in the urine of healthy controls grouped by age **(A)** or age and sex **(B)**. Neopterin levels were assessed in urine samples collected in the morning with a minimum of 2 h retention in a collector plastic cup. Neopterin quantification was performed by liquid chromatography coupled with fluorescent detection as previously reported by our group (Scheffer et al., 2019). Graph was generated with GraphPad Software version 9.

Finally, Lunetta's work was a pioneer in demonstrating that systemic inflammation can be followed in urine samples of ALS-affected individuals. In addition, the authors stressed that this measurement might become a useful surrogate endpoint for classifying ALS candidates for future drugs aimed at intervening in the chronically exacerbated inflammatory response characteristics of ALS.

## Author Contributions

Material preparation and data collection were performed by GE and AL. The biochemical analyses were performed by DS. The first draft of the manuscript was written by AL. All authors contributed to the study conception and design, read, and approved the final manuscript.

## Conflict of Interest

The authors declare that the research was conducted in the absence of any commercial or financial relationships that could be construed as a potential conflict of interest.
